# Supplementary material for: Content-rich biological network constructed by mining PubMed abstracts
Source: BMC Bioinformatics. 2004 Oct 8;5:147. doi: 10.1186/1471-2105-5-147 (PMC528731; doi:10.1186/1471-2105-5-147)
Supplement: Additional File 5 — The original Chilibot query results of the term "long-term potentiation (LTP)" and 22 other terms, limiting the latest references analyzed to the years 1990, 1995, 2000, and 2004. [file 1471-2105-5-147-S5.bz2 › chilibotAdditionalFile5/ltp1995/html/ATF_CAMKII.html]

 


 **ATF** and **CAMKII** 
  
Found 1 abstracts in PubMed,  **1 abstracts were retrieved and analyzed**.  


---

 Search Google  |
 PDF files only 
|  EDU domain only 

---

- Mol Cell Biol, 1994   **Calcium calmodulin dependent protein kinase types II and IV differentially regulate CREB dependent gene expression.** .
  Phosphorylation of CREB cyclic AMP cAMP response element CRE binding protein by cAMP dependent protein kinase PKA leads to the activation of many promoters containing CREs.
  In neurons and other cell types, CREB phosphorylation and activation of CRE containing promoters can occur in response to elevated intracellular Ca2ion .
  In cultured cells that normally lack this Ca2ion responsiveness, we confer Ca 2ion mediated activation of a CRE containing promoter by introducing an expression vector for Ca2ion calmodulin dependent protein kinase type IV CaMKIV .
  Activation could also be mediated directly by a constitutively active form of CaMKIV which is Ca2ion independent.
  The CaMKIV mediated gene induction requires the activity of CREB **ATF** family members but is independent of PKA activity.
  In contrast, transient expression of either a constitutively active or wild type Ca2ion calmodulin dependent protein kinase type II **CaMKII** fails to mediate the transactivation of the same CRE containing reporter gene.
  Examination of the subcellular distribution of transiently expressed CaMKIV and **CaMKII** reveals that only CaMKIV enters the nucleus.
  Our results demonstrate that CaMKIV, which is expressed in neuronal, reproductive, and lymphoid tissues, may act as a mediator of Ca 2ion dependent gene induction.
